# Production and Quality Characteristics of Starter Culture Fermented Aliha, a Maize-Based Indigenous Fermented Beverage

**DOI:** 10.1155/ijfo/6665795

**Published:** 2025-06-24

**Authors:** Felix Kwashie Madilo, Emmanuel Letsyo, Nii Korley Kortei, Otilia Abla Adzinyo, Angela Parry-Hanson Kunadu

**Affiliations:** ^1^Food Science and Technology Department, Ho Technical University, Ho, Ghana; ^2^Nutrition and Dietetics Department, School of Allied Health Sciences, University of Health and Allied Sciences, Ho, Ghana; ^3^Hospitality and Tourism Management Department, Ho Technical University, Ho, Ghana; ^4^Nutrition and Food Science Department, University of Ghana, Accra, Ghana

**Keywords:** corn, fermentation, nutritional quality, probiotics, safety, spontaneous

## Abstract

Probiotics are beneficial microorganisms that are used as starter cultures to improve the safety, sensory qualities, and nutritional properties of the final products. Therefore, this study was designed to use selected lactic acid bacteria (LABs) and yeast strains isolated from spontaneously fermented *Aliha* to improve the quality of the final *Aliha*. Four LAB strains (*Limosilactobacillus fermentum* Cm1-24-L1a, *Limosilactobacillus fermentum* UL, *Limosilactobacillus fermentum* TCD45.2, and *Limosilactobacillus fermentum* NBRC 15885) and two yeast strains (*Pichia kudriavzevii* CY902 and *Pichia kudriavzevii* CBS 5147 18) were sourced and used to ferment corn malts into probiotic *Aliha*. The pH, acidity, growth rates, hygiene conditions, nutritional qualities, and sensory properties were determined. The results revealed that strain NBRC 15885 recorded the lowest pH value (2.67) and the highest acid values of 0.71% after 24 h of fermentation. While strain CBS 5147 18 recorded the highest viability rate (4.30 log CFU/mL) after 24 h of fermentation, *Aliha* fermented by strain UL recorded the second lowest indicator counts for aerobic plate count (APC), Enterobacteriaceae, and fungi, making it the safest. The most nutritious (carbohydrates, crude fibre, proteins, potassium, calcium, and magnesium) *Aliha* was produced by strains NBRC 15885 and TCD45.2 and the least by strain CY902. However, *Aliha* fermented by strain UL was highly accepted by the assessors as it recorded the highest values of appearance (4.56), color (4.39), taste (4.63), and overall acceptability (4.99). Hence, the use of defined starter cultures has improved the quality parameters of the beverages produced and therefore must be recommended to the local producers for safer and better *Aliha* production.

## 1. Introduction

Foods and beverages made from fermented cereals are rich in dietary fibers and minerals [1]. Cereals in their natural state possess dietary fibers, proteins, vitamins, minerals, antioxidants, and other phytochemicals [2–4]. Due to flat flavor and poor organoleptic qualities, fermentation is recommended to improve the sensory and nutritional qualities and to prevent the existence of spoilage microbiota in the final fermented products [5]. Cereals can be used as fermentable substrates for the growth of probiotics [6, 7]. Several studies have published the importance of consuming cereal-based fermented foods and beverages, including the reduction of cardiovascular problems, diabetes, and cancer [8–10]. *Aliha* is a spontaneous maize-based fermented beverage [11]. It is produced through the process of initial washing of the corn, followed by soaking, malting, drying, milling, the addition of cold or warm water, boiling, straining, fermentation (3 days), and caramelization for the unique flavor and taste desired by the patrons.

Fermentation is a natural means of improving the nutritional values of the raw materials while preventing the detrimental elements of the final products [12]. The process solubilizes the phytate complex and reduces antinutrient contents, thereby improving the bioavailability of nutrients, essential amino acid contents, total soluble solids, and nonprotein nitrogen contents [10]. The fermentation process has also been proven to better the sensory and antioxidant properties of various cereals [8].

Studies have shown that the consumption of cereal-based fermented foods and beverages not only extends the shelf-life and improves the acceptability but also enhances the organoleptic properties and digestibility of the final product [3, 13]. Despite these benefits, however, natural fermentation (uncontrolled fermentation) or backsloping results in poor qualities of sensory and nutritional properties, inconsistency in the quality of the final products, risks of batch failure, and has a lot of safety concerns [14, 15]. Hence, the use of starter cultures with probiotic potential is recommended and encouraged for several reasons. Starter cultures to be considered for controlled fermentation must possess certain technological and probiotic qualities such as being safe for human consumption, high fermentability properties, improved sensory and nutritional qualities, high antagonistic abilities, and high bioactive compounds production capacity [16–18]. Wuyts et al. [19] stated that lactic acid bacteria (LABs) and yeast strains can be selected from indigenous fermented food products as starters to standardize fermented cereal products when their probiotic, sensory, and technological potentials are evaluated. Properly selected probiotics can produce sufficient bioactive compounds to inhibit spoilage microflora, improve intestinal microbes, and showcase immunomodulatory, antidiabetic, and anticarcinogenic potentials [12, 20]. Hence, this study is needed because *Aliha*, a naturally produced beverage, has issues such as short shelf-life, poor organoleptic, and nutritional properties [11]. This study used LABs and yeast strains previously isolated from *Aliha* to produce *Aliha* with enhanced safety, sensory, and nutritional qualities.

Tsafrakidou et al. [12] have shown that LABs and yeast species have been used as starter cultures to produce food and beverages. These microflora include genera such as *Lactobacillus*, *Leuconostoc*, *Pediococcus*, *Bifidobacterium*, and *Enterococcus* [21]. They produce compounds such as organic acids (lactic acids, acetic acid, H_2_O_2_, CO_2_, etc.), antimicrobial substances, and bioactive peptides [3]. These antimicrobial compounds and other bacteriocins and potential functional bioactive substances prevent the existence of spoilage and pathogenic microbes, thereby extending the shelf-life of the final products and also assuring the safety of the consumers [22, 23]. The probiotics can resolve gastrointestinal issues; serve as anticholesteremic, immunomodulatory, antidiabetic, anti-inflammatory, antiobesity, and antioxidant [16, 22, 24]; and improve mental health difficulties and intestinal microflora [25]. They also treat diseases including lactose intolerance, antibiotic-induced diarrhea, infantile diarrhea, travelers' diarrhea, constipation, colon cancer, hypercholesterolemia, and vaginitis [24, 26]. Therefore, the main objective of this study was to produce *Aliha* with extended shelf-life and enhanced organoleptic and nutritional properties.

## 2. Materials and Methods

### 2.1. Source of the Starter Cultures and Initial Preparation

The four LABs and two yeast starter cultures (Table 1) were sourced from the Microbiology Laboratory of the Nutrition and Food Science Department, University of Ghana. The starter cultures, which were kept in MRS broth and malt extract broth (MEB) containing 20% (*v*/*v*) glycerol for LABs and yeasts, respectively, were revived for the current study using the method by Vieira-Dalodé et al. [27] with some modifications. Briefly, a loopful of LABs and yeast cultures was streaked on MRS agar (Oxoid) and malt extract agar (MEA) (HiMedia), respectively, and the plates were incubated at 30°C for 24 h for LAB and at 25°C for 48 h for yeast. A single visible colony from individual plates was then transferred into 10 mL of MRS broth and MEB. After the cultures were incubated overnight at 28°C, about 100 *μ*L of each of the cultures was transferred into their corresponding tubes containing 10 mL of each of the media and incubated overnight at 28°C. The cultures were centrifuged at 3500 × g for 15 min, and the pellets were washed twice in 10 mL of sterile peptone water and stored in sterile peptone water to give concentrations of about 10^6^ and 10^4^ cells/mL for LAB and yeast, respectively.

### 2.2. Production of Starter Culture Fermented *Aliha*

The wort was produced traditionally [11] by the local producers. Briefly, malt was milled and soaked overnight. More water was added, and the mixture was transferred into a 20 L cooking pot. The mixture was boiled until there was no foam, and the mixture was allowed to cool at ambient temperatures (20°C–25°C). The mixture was strained twice, and about 1000 mL was distributed into 2 L sterile containers. Subsequently, while about 0.5% (by volume) of each of the inocula (LAB and yeast) was inoculated and incubated at ambient temperature for 24 h, the control (spontaneous) was allowed to ferment for 72 h. These were prepared for microbiological, nutritional, and sensory quality analyses. Again, both starter culture and spontaneously fermented beverages were withdrawn at 0, 6, 12, 18, and 24 h for the analysis of pH, brix, and lactic acid production. The microbial, pH, and total titratable acidity (TTA) analyses were carried out in the laboratory of the Food Science and Technology Department of Ho Technical University. The final starter culture and spontaneously fermented samples were stored at −18°C for nutritional and sensory characterization.

### 2.3. pH and Titratable Acidity

After the digital pH meter (Crison Instrument, Barcelona) was calibrated using hydrochloric acid (4.0) and sodium hydroxide (7.0), the pH of the samples was measured. The TTA was determined by titrating 10 mL of the sample against NaOH and phenolphthalein as an indicator. The result was expressed as lactic acid.

### 2.4. Proximate Mineral Analysis

The concentration of both proximate and mineral in both starter culture and spontaneously fermented Aliha was evaluated using the Official Methods of Analysis of the Association of Official Analytical Chemists (AOAC) International [28]. The AOAC Method No. (925.10) was employed to determine the moisture contents. The Kjeldahl method (AOAC Method No. 978.04) was used to determine the crude protein content, while the crude fat content was determined by exhaustively extracting a known weight of each CF in petroleum ether (boiling point, 40°C–60°C) using a Soxhlet extractor (AOAC Method No. 930.09). The ash content was determined in a muffle furnace at 550°C (AOAC Method No. 930.05). The enzymatic-gravimetric method (AOAC Method No. 930.10) was employed to determine the crude fiber content. The carbohydrate content was calculated by difference (subtracting the sum of the percentages of moisture, fat, crude protein, ash, and crude fiber from 100%.).

For mineral concentration, the samples were subjected to ashing at 550°C, followed by boiling with 10 mL of 20% hydrochloric acid in a beaker. The resulting solution was then filtered into a 100 mL standard flask and diluted with deionized water to reach the mark. To determine the levels of calcium (Ca), iron (Fe), magnesium (Mg), potassium (K), copper (Cu), and zinc (Zn) in the solution, the atomic absorption spectrometry method was employed. For absorbance measurements, a Perkin 400 atomic absorption spectrometer (Perkin Elmer Analyst 400, Waltham, MA, United States) equipped with an air/acetylene flame and respective hollow cathode lamps was used. The mineral content results were expressed as milligrams per 100 g. The samples were analyzed in duplicates.

### 2.5. Microbiological Analysis

#### 2.5.1. Proliferation of Microbial Starters in Fermented *Aliha* for pH, Lactic Acid, and Brix

The growth rates of microbial starter cultures used in this study were evaluated. The wort was prepared, and 5 L was distributed into each of the 10 L sterile containers. The samples were then inoculated with the purified cells of LABs and yeasts and distributed in 500 mL bottles before being incubated at an ambient temperature. During the incubation, the samples were variously withdrawn at 0, 6, 12, 18, and 24 h. The withdrawn samples were serially diluted, and about 1 mL of each dilution was pour-plated on MRS agar for LAB and on YEA (yeast extract agar) for yeast. The inoculated plates were incubated anaerobically at a temperature of 30°C for 24–48 h for LABs and aerobically between the hours of 24–72 at 30°C for yeasts. At the end of the incubation periods, the plates with visible colonies between 25 and 250 were enumerated and recorded.

#### 2.5.2. Hygiene Indicator Analysis

The hygiene indicators such as aerobic plate count (APC), Enterobacteriaceae, fecal coliform (FC), staphylococci, and fungi were used to evaluate the final beverages to determine the integrity and the pathogenic bacterial inhibitory ability of the starter cultures used to ferment the products. For each sample, fourfold serial dilutions were prepared. About 100 *μ*L of each dilution was pour-plated on plate count agar for APC, MacConkey agar supplemented with glucose for Enterobacteriaceae, violet red bile glucose agar (VRBGA) for FC, mannitol salt agar (MSA) for staphylococci, and MEA for yeasts and molds (fungi) in duplicate. The PCA, MacConkey, and VRBGA were incubated at 37°C for 24–48 h [29]. The MEA inoculated plates were incubated at 25°C for 72 h. The plates were then observed, the colonies were counted, and the results were recorded.

#### 2.5.3. Sensory Evaluation of the Probiotic *Aliha*

The method by Hassen et al. [30] was used with slight modifications for the consumer acceptability test. The chilled starter culture and spontaneously fermented samples were variously presented to 50 (29 males and 21 females) randomly selected untrained panels but regular consumers of *Aliha* for tasting. The test was carried out between 1:00 and 3:00 pm, which are the best times the beverage is mostly consumed. A 5-point hedonic scale (5 = *like extremely*; 4 = *like very much*; 3 = *like moderately*; 2 = *like slightly*; 1 = *neither like nor dislike*; 4 = *dislike slightly*; 3 = *dislike moderately*; 2 = *dislike very much*; 1 = *dislike extremely*) was used to determine the acceptability level of the different treatment characteristics (aroma, taste, color, appearance, and overall acceptability) of the samples. The samples were blind-labeled with randomized three-digit codes and presented to the panelists randomly to avoid bias. The samples were served in transparent plastic cups, together with bottled water (Voltic) at room temperature. The water was to rinse the palate before and between tastings to prevent carryover tastes. All the various experimental analyses were carried out in duplicates. Ethical approval was granted (HTU/DRI/EC2024-035) by the ethical committee of the Directorate of Research and Innovation (DRI), Ho Technical University.

### 2.6. Statistical Analysis

All data obtained were subjected to analysis of variance (ANOVA) IBM SPSS Version 25.0. The comparisons of mean changes in microbial counts, pH, and TTA and their significance differences were obtained using descriptive statistics and one-sample *t*-test, respectively. The figures were obtained using Microsoft Excel, 2013.

## 3. Results and Discussion

### 3.1. Physicochemical Properties of Starter Culture Fermented *Aliha*

**Figure 1:**
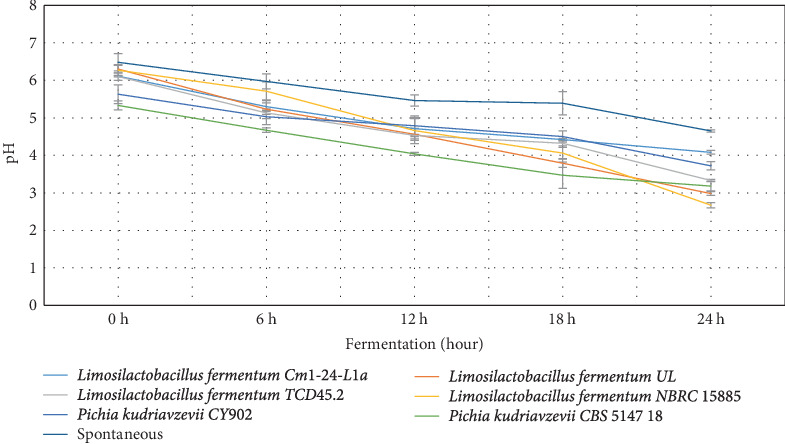
The proliferation of the microbial fermenters in the pH of the fermented Aliha samples.

**Figure 2:**
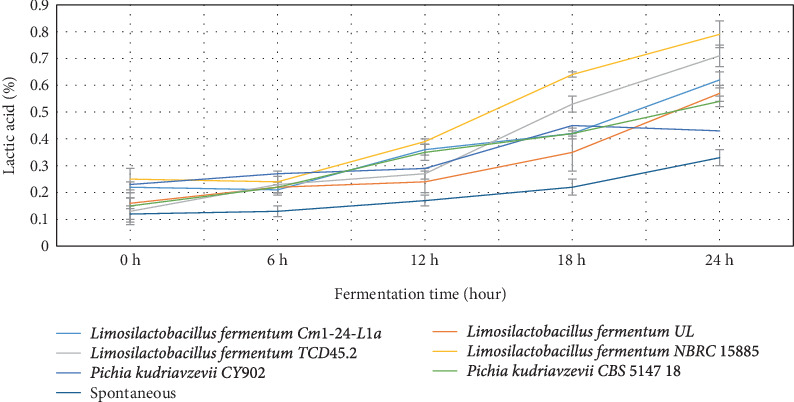
The trend of acid produced by the various starter cultures.

**Figure 3:**
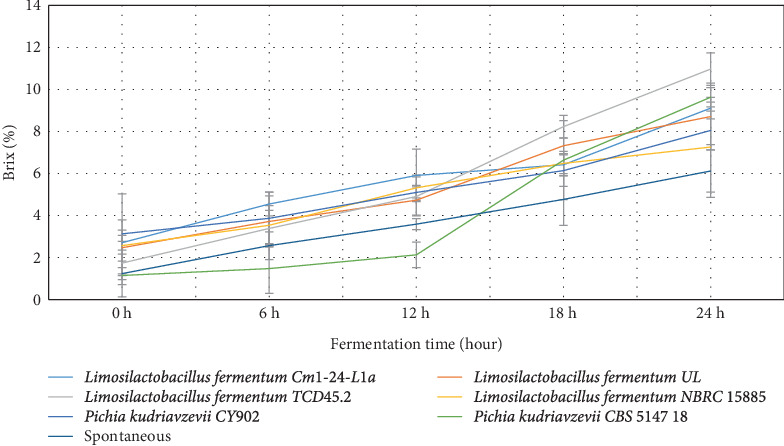
Quantity of brix produced by the starter cultures.

The behavior of the pH, lactic acid, and brix in both starter culture and spontaneously fermented *Aliha* were determined, and the findings are summarized in Figures 1, 2, and 3.  Figure 1 indicates that after 12 h of fermentation, there was a gradual reduction of the pH of each of the samples across the fermenting starters. However, *Limosilactobacillus fermentum* NBRC 15885 recorded the lowest value at the end of the 24 h of fermentation, ranging from 6.27 to 2.67, followed by *Limosilactobacillus fermentum* UL (6.30–2.98) with *Limosilactobacillus fermentum* Cm1-24-L1a recording the highest pH values (6.11–4.08). Consequently, the decrease in pH contributes to the increase in lactic acid production. This is very evident in Figure 2. This is so because, during fermentation, the microbial fermenters involved in the fermentation produce a large amount of organic compounds including lactic acid in the fermenting medium, which lowers the pH of the final fermented products [31]. The figure reveals that there was a sharp increase in the lactic acid produced by most of the starters, particularly after 12 h of fermentation. Specifically, *Limosilactobacillus fermentum* NBRC 15885 had the sharpest increase from 0.25% to 0.79% within 24 h. This was followed by *Limosilactobacillus fermentum* TCD45.2 (0.13%–0.71%), with the lowest being *Pichia kudriavzevii* CY902 (0.23%–0.43%). Comparatively, the total acids produced by the naturally fermented *Aliha* at the end of the third day were the lowest (0.22%). This means that the rate of growth of the microbial fermenters in the naturally fermented *Aliha* was lower as compared to *Aliha* fermented with defined starter cultures. This is an excellent performance by the fermenting starters because high lactic acid production is required for the reduction of fermentation periods, the rates of microbial pathogenic contamination, the improvement of the sensory and nutritional properties, and the extension of the shelf-life of the final beverage. Lactic acids and other metabolites are produced when microorganisms (LABs) consume the available carbohydrates (glucose) through glycolysis. They convert glucose into pyruvate, which is then reduced to lactic acids [31, 32].

For brix (Figure 3), the highest quantities range from 1.74% to 10.97% for *Limosilactobacillus fermentum* TCD45.2, followed by *Pichia kudriavzevii* CBS 5147 18 (1.15%–9.63%). The least brix values were recorded by *Limosilactobacillus fermentum* NBRC 15885 (2.57%–7.26%). The quantity of brix produced in the fermented medium contributes to the quality of the sensory and nutritional values of the final beverage.

The level of pH recorded by the current study was similar to that of *Boza*, *Gowé*, *Kunun-zaki*, *Mahewu*, and *Obushera* [11, 33]. Ziarno et al. [34] revealed that the rise in pH determines the amount of organic acid produced in the fermenting medium which is important to ensure the quality of the final product. Mora-Villalobos et al. [35] stated that the level of pH and lactic acid coupled with the production of high organic acids such as fatty acid, bacteriocin, hydrogen peroxide, and peptides serve as inhibitors against antagonistic microbes for the product's safety and shelf-life extension. The higher the population of LABs and yeasts in the fermenting medium, the higher the organic acid they produce in the medium. The organic acids are important for enhancing the organoleptic and sensory qualities of the final products. Again, the higher the organic acids, the lower the microbial pathogens and the extended shelf life of the final products [11].

### 3.2. Microbiological Analysis

The results of the microbial growth rate and hygiene indicator analyses of both starter culture and spontaneously fermented *Aliha* samples are presented in Tables 2 and 3. The growth rates of the starter cultures used in this study were determined to assess their viability across the sampling points. The results (Table 2) show that *Aliha* fermented by strains CBS 5147 18 (3.93 log CFU/mL), NBRC 15885 (3.96 log CFU/mL), CBS 5147 18 (4.11 log CFU/mL), and CBS 5147 18 (4.30 log CFU/mL) recorded the highest viability rates at 6, 12, 18, and 24 h of fermentation, respectively. At the same collection point, strains Cm1-24-L1a (3.39 log CFU/mL), UL (3.05 log CFU/mL), Cm1-24-L1a (3.62 log CFU/mL), and NBRC 15885 (4.24 log CFU/mL) registered the lowest growth viability rates. However, there were no significant statistical differences (*p* ≤ 0.05) among the counts across the sampling points.

One of the significant benefits of the use of starter cultures for fermentation is to reduce the fermentation periods, in that case, from 72 to 24 h or lower for *Aliha*. The results (Table 2) show that after 24 h of fermentation, the viability of strain CBS 5147 18 increased from 3.65 to 4.30 log CFU/mL, followed by strains TCD45.2 from 3.29 to 4.29 log CFU/mL and CY902 (3.42–4.29 log CFU/mL), with the least being strain NBRC 15885 from 3.78 to 4.24 log CFU/mL. The growth of LABs in the fermented foods and beverages is a result of favorable conditions such as specific temperature ranges, pH levels, abundance of fermentable sugars, and the absence of oxygen [31]. Again, their dominance could also be influenced by salt concentration and the absence of pathogenic microorganisms [36]. This is an indication that when our starters are used for controlled fermentation, they would multiply faster and produce sufficient bioactive compounds for safety, thereby improving the sensory and nutritional qualities of the final beverages.

The hygienic condition of the starter culture fermented *Aliha* was also analyzed to ensure the safety of the final product. At the end of the study, the results (Table 3) revealed that samples fermented by strains Cm1-24-L1a, CBS 5147 18, TCD45.2, TCD45.2, and CY902 recorded the lowest APC (2.67 log CFU/mL), Enterobacteriaceae (2.67 log CFU/mL), FC (2.76 log CFU/mL), fungi (2.44 log CFU/mL), and enterococci (2.83 log CFU/mL) counts, which signify the safety of the products. However, the most contaminated probiotic fermented samples were TCD45.2 (3.10 log CFU/mL), CY902 (2.95 log CFU/mL), CY90 (3.39 log CFU/mL), CBS 5147 18 (3.05 log CFU/mL), and CBS 5147 18 (3.14 log CFU/mL) for APC, Enterobacteriaceae, FC, fungi, and enterococci, respectively. Comparatively, even the highly contaminated starter culture fermented samples were safer than the spontaneously fermented samples. More importantly, the majority of the starter culture fermented samples were within the thresholds. However, there exists no statistically significant difference among the counts across the microbial indicators except for the sample fermented with strain TCD45.2. The significant reduction of the spoilage and pathogenic microbiota means that the microbial metabolites such as lactic acids, acetic acids, ethanols, carbon dioxide, hydrogen peroxides, and nisin produced by the probiotics in the fermented media were sufficient enough to inhibit the presence of these pathogens.

Tsafrakidou et al. [12] reported that the probiotic organisms employed in the fermentation process produce organic compounds with better sensory and nutritional qualities, which prevent the presence of microbial pathogens. The metabolic compounds produced significantly reduce the values of pH, thereby creating an acidic medium that prevents the growth of microbial pathogens, hence extending the shelf life of the final product [12]. Adebiyi et al. [37] and Juodeikiene et al. [38] stated that LABs and yeasts can produce ethanol, hydrogen peroxide, and secondary organic compounds as inhibitory compounds for the growth of microbial pathogens. Again, *Limosilactobacillus fermentum* FUA 3321 used to produce Ting was able to reduce the presence of mycotoxins by 98% [39].

### 3.3. Nutritional Composition

The findings of the nutritional compositions of the starter culture and spontaneously fermented *Aliha* are shown in Table 4. From the table, the highest carbohydrate (5.93%) and crude fiber values were registered by strain NBRC 15885, proteins (4.44) by strain TCD45.2, crude fiber (1.06%) by NBRC 15885, ash (0.44%) by CBS 5147 18S, and crude fats (0.50%) by strain CY902. For minerals, the highest K (183.4) value was presented by strain NBRC 15885, Mg (63.61 mg/100) by strain Cm1-24-L1a, Cu (2.52 mg/100) by UL, Ca (1827.5 mg/100) by TCD45.2, Fe (3.58 mg/100) by UL, and Zn (0.56 mg/100) by strain TCD45.2. Moreover, the most nutritious starter culture fermented *Aliha* was produced by strains NBRC 15885 and TCD45.2, and the least was produced by strain CY902. Comparatively, all the starter culture-fermented *Aliha* were more nutritious than the spontaneously fermented ones. However, there exist statistically significant differences (*p* ≤ 0.05) among the nutritional values across the samples.

Cereal grains are major sources of proximates and minerals including vitamins [12, 40]. The quantities of the current probiotic *Aliha* were far better than all five spontaneously fermented *Aliha* reported by Madilo et al. [11]. The significant increase in proximates and minerals recorded from this study was a result of the sufficient metabolites produced by the probiotics which were able to hydrolyze every bit of the grain, even the undigestible oligosaccharides, by increasing the surface area for further fermentation [40].

Ignat et al. [33] reported that the proximates, minerals, and other essential components of *Oshikundu*, a Namibia beverage that was fermented with *Limosilactobacillus* species, including *fermentum*, were significantly improved. Madilo et al. [40] indicated that probiotics are used to enhance the bioavailability of macro- and micronutrients of cereal-based fermented beverages.

### 3.4. Sensory Properties

**Figure 4:**
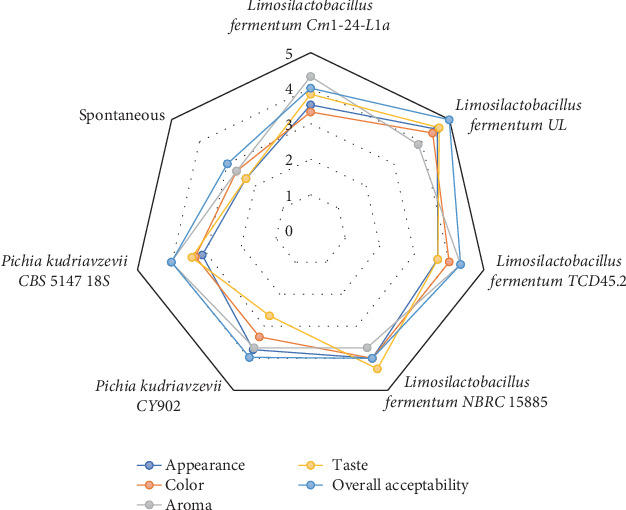
Performance of sensory properties of starter culture fermented Aliha as against naturally fermented Aliha.

The sensory analysis was conducted to determine the appreciable level of sensory parameters contributed to the fermented products by the fermenting microbes. The results (Figure 4) revealed that the organoleptic characteristics of the *Aliha* fermented by strain UL were highly accepted as it recorded the highest values of appearance (4.56), color (4.39), taste (4.63), and overall acceptability (4.99). The least accepted beverage was fermented by strain CY902 for aroma (3.67) and taste (2.67) and strain CBS 5147 18S for appearance (3.13) and color (3.33). However, the sensory parameters of the spontaneously fermented *Aliha* were heavily rejected by the panelists. The sensory qualities produced by strain UL were not surprising because of its ability to produce high amounts of organic acids and other metabolites that were able to break down even the undigestible parts of the grain. The foul or flat flavor and aroma compounds produced by the pathogenic and spoilage microbiota that cloud the sensory parameters might have been prevented by this strain (as it was recorded as the second safest beverage), hence improving the sensory properties.

Intentionally incorporating LABs and yeasts is one of the best techniques for producing cereal-based beverages with desired organoleptic properties [34, 41]. The improvement of sensory parameters in cereal-based probiotic fermented food and beverages might be due to the contribution of microbial metabolites (volatile compounds) and nonvolatile compounds such as sugar and carboxylic acid by bacterial and yeast starters, which improve the taste and aroma of the final products [12, 33, 40].

## 4. Conclusion

The starter cultures (*Limosilactobacillus fermentum* Cm1-24-L1a, *Limosilactobacillus fermentum* UL, *Limosilactobacillus fermentum* TCD45.2, *Limosilactobacillus fermentum* NBRC 15885, *Pichia kudriavzevii* CY902, and *Pichia kudriavzevii* CBS 5147 18) considered for the study were able to produce better *Aliha* than the naturally fermented one. The organoleptic properties and the nutritional and microbial qualities of the starter culture fermented beverages were more enhanced and appreciated compared to those fermented spontaneously. Specifically, the samples fermented by starter strains such as Cm1-24-L1a, CBS 5147 18, TCD45.2, TCD45.2, and CY902 recorded the lowest hygiene indicators (APC, Enterobacteriaceae, FC, fungi, and enterococci) signifying the safety of the beverages. Moreover, the most nutritious starter culture fermented *Aliha* was produced by strains NBRC 15885 and TCD45.2. Finally, *Aliha* fermented by the UL strain was highly accepted as it recorded the highest values of appearance, color, taste, and overall acceptability. However, the study recommends that further studies should be conducted to identify the various organic acids produced in the beverage by the individual defined cultures. These metabolites can be kept and used in the absence of the probiotic strains.

## Figures and Tables

**Table 1:** Microbial starters considered for this study.

**SN**	**Organism**	**Strain**
1	*Limosilactobacillus fermentum*	Cm1-24-L1a
2	*Limosilactobacillus fermentum*	UL
3	*Limosilactobacillus fermentum*	TCD45.2
4	*Limosilactobacillus fermentum*	NBRC 15885
5	*Pichia kudriavzevii*	CY902
6	*Pichia kudriavzevii*	CBS 5147 18

**Table 2:** Proliferation of the microbial starter cultures in the fermenting medium (log CFU/mL).

**Strain**	**0 h**	**6 h**	**12 h**	**18 h**	**24 h**	**p** ** value**
Cm1-24-L1a	2.96 ± 0.16^c^	3.39 ± 0.35^bc^	3.85 ± 0.06^a^	3.62 ± 0.49^b^	4.24 ± 0.06^a^	0.002
UL	3.31 ± 0.15^c^	3.89 ± 0.15^b^	3.05 ± 0.03^d^	4.05 ± 0.03^b^	4.25 ± 0.02^a^	0.001
TCD45.2	3.29 ± 0.18^d^	3.74 ± 0.06^c^	3.93 ± 0.04^b^	4.05 ± 0.06^b^	4.29 ± 0.11^a^	0.001
NBRC 15885	3.78 ± 0.01^b^	3.91 ± 0.05^b^	3.96 ± 0.04^b^	3.91 ± 0.22^b^	4.24 ± 0.14^a^	0.009
CY902	3.42 ± 0.10^c^	3.83 ± 0.04^bc^	3.67 ± 0.53^bc^	3.99 ± 0.02^ab^	4.29 ± 0.05^a^	0.015
CBS 5147 18	3.65 ± 0.19^b^	3.93 ± 0.05^ab^	3.65 ± 0.55^b^	4.11 ± 0.05^ab^	4.30 ± 0.02^a^	0.051

*Note:* Means that do not share a letter on a roll are significantly different (*p* = 0.05).

**Table 3:** Values of hygienic indicator of 24 h starter culture fermented and 72 h naturally fermented Aliha.

**Indicator**	**APC**	**Enterobacteriaceae**	**Fecal coliform**	**Fungi**	**Enterococci**	**p** ** value**
Cm1-24-L1a	2.67 ± 0.55^a^	2.89 ± 0.09^a^	2.95 ± 0.09^a^	2.98 ± 0.04^a^	2.97 ± 0.09^a^	0.584
UL	2.79 ± 0.03^a^	2.71 ± 0.61^a^	2.94 ± 0.10^a^	2.86 ± 0.12^a^	2.87 ± 0.03^a^	0.767
TCD45.2	3.10 ± 0.15^a^	2.86 ± 0.16^ab^	2.76 ± 0.11^b^	2.44 ± 0.28^c^	2.87 ± 0.09^ab^	0.011
NBRC 15885	2.92 ± 0.15^a^	2.78 ± 0.24^a^	2.87 ± 0.09^a^	2.87 ± 0.11^a^	2.86 ± 0.08^a^	0.837
CY902	2.95 ± 0.07^a^	2.95 ± 0.03^ab^	3.39 ± 0.53^ab^	3.04 ± 0.04^b^	2.83 ± 0.16^ab^	0.148
CBS 5147 18	2.95 ± 0.07^a^	2.67 ± 0.47^ab^	3.00 ± 0.05^ab^	3.05 ± 0.07^b^	3.14 ± 0.12^ab^	0.185
Control (72 h)	3.09 ± 0.11^a^	3.13 ± 0.12^b^	3.10 ± 0.17^b^	3.08 ± 0.09^b^	3.36 ± 0.08^b^	0.074
AL	4.00	3.00	2.00	3.00	3.00	

*Note:* Means that do not share a letter on a roll are significantly different (*p* ≤ 0.05).

Abbreviation: AL, acceptable limit (GSA CDGS 955: 2018).

**Table 4:** Proximate and mineral compositions of the probiotic Aliha against naturally fermented Aliha.

**Variable**	**Spontaneous**	**Cm1-24-L1a**	**UL**	**NBRC 15885**	**CBS 5147 18S**	**CY902**	**TCD45.2**	**p** ** value**
Proximate content (%)								
Moisture	96.05 ± 0.09^a^	90.11 ± 0.01^e^	90.26 ± 0.07^d^	89.23 ± 0.02^f^	90.38 ± 0.08^c^	91.43 ± 0.03^b^	91.38 ± 0.02^b^	0.001
Carbohydrates	3.06 ± 1.00^c^	5.31 ± 0.01^a^	4.53 ± 0.32^bc^	5.93 ± 0.03^a^	4.40 ± 0.04^c^	4.72 ± 0.58^bc^	3.06 ± 0.06^d^	0.001
Proteins	0.61 ± 0.10^a^	3.72 ± 0.02^d^	4.13 ± 0.15^d^	3.21 ± 0.21^e^	4.10 ± 0.11^c^	3.05 ± 0.05^d^	4.44 ± 0.14^a^	0.001
Crude fiber	0.09 ± 0.09^d^	0.54 ± 0.04^bc^	0.73 ± 0.07^b^	1.06 ± 0.06^a^	0.49 ± 0.09^c^	0.41 ± 0.30^c^	0.52 ± 0.03^bc^	0.001
Ash	0.05 ± 0.02^d^	0.14 ± 0.00^c^	0.26 ± 0.10^b^	0.25 ± 0.05^b^	0.44 ± 0.04^a^	0.24 ± 0.01^b^	0.41 ± 0.01^d^	0.001
Crude fats	0.14 ± 0.10^cd^	0.18 ± 0.01^d^	0.41 ± 0.05^b^	0.32 ± 0.02^c^	0.19 ± 0.00^d^	0.50 ± 0.04^a^	0.17 ± 0.01^d^	0.001
Mineral content (mg/100)								
Potassium	97.55 ± 1.07^c^	181.65 ± 10.30^a^	142.63 ± 9.42^b^	183.4 ± 9.82^a^	150.97 ± 12.44^b^	141.9 ± 0.00^b^	1.44 ± 0.15^d^	0.001
Magnesium	27.65 ± 0.06^d^	63.61 ± 3.10^a^	57.24 ± 8.89^b^	44.09 ± 3.95^c^	55.45 ± 2.33^b^	44.45 ± 3.99^c^	60.67 ± 1.30^a^	0.001
Copper	1.26 ± 0.46^b^	2.07 ± 0.02^ab^	2.52 ± 0.81^a^	2.13 ± 0.95^b^	2.45 ± 0.03^a^	2.16 ± 1.01^ab^	1.18 ± 0.10^b^	0.111
Calcium	88.88 ± 1.72^d^	263.18 ± 10.05^b^	159.30 ± 17.70^c^	181.6 ± 0.00^c^	174.22 ± 0.91^c^	124.55 ± 10.20^cd^	1827.5 ± 100.10^ab^	0.001
Iron	1.18 ± 0.69^c^	2.3 ± 0.00^abc^	3.58 ± 1.04^a^	2.14 ± 0.10*b*^c^	2.48 ± 1.05^abc^	2.37 ± 0.87^abc^	2.75 ± 0.98	0.078
Zinc	0.35 ± 0.275^ab^	0.39 ± 0.29^ab^	0.52 ± 0.15^a^	0.34 ± 0.05^ab^	0.41 ± 0.07^ab^	0.56 ± 0.19^a^	0.17 ± 0.12^b^	0.263

*Note:* Means that do not share a letter in a roll are significantly different (*p* ≤ 0.05).

## Data Availability

Data sharing is not applicable to this article as no new data were created or analyzed in this study.
